# Imaging and clinical features of Castleman Disease

**DOI:** 10.1186/s40644-019-0238-0

**Published:** 2019-07-25

**Authors:** Shuang Zhao, Ying Wan, Zixing Huang, Bin Song, Jianqun Yu

**Affiliations:** 1 Department of Radiology, West China Hospital, Sichuan University, No. 37, GUOXUE Alley, Chengdu, 610041 China; 2 Department of Pathology, West China Hospital of Sichuan University, Chengdu, 610041 China

**Keywords:** Castleman disease, Computed tomography, Magnetic resonance imaging, Diffusion-weighted imaging

## Abstract

**Background:**

Castleman disease (CD) is a group of uncommon lymphoproliferative disorders that is easily confused with lymphoma or other solid tumors. The purpose of our study was to evaluate the imaging and clinical findings of CD, and thus improve the understanding and diagnosis of CD.

**Methods:**

This retrospective study included 74 patients (37 men and 37 women, mean age ± standard deviation, 35 ± 15.2 years,) with histopathologically confirmed CD diagnosed based on CT or MRI between January 2010 and May 2017. The CT and MRI findings were analyzed by two radiologists in consensus, and clinical presentation and histopathologic characteristics were documented.

**Results:**

The CD subtypes included 61 hyaline vascular variant cases (82.4%) and 13 plasma cell variant cases (17.6%). Unicentric CD and multicentric CD were observed in 65 (87.8%) and 9 (12.2%) patients, respectively. On non-enhanced CT, enlarged nodes with hypodensity or isodensity were seen, whereas varying degrees of enhancement were observed in contrast-enhanced CT. Homogeneous and heterogeneous enhancements were observed in 43 (62.3%) and 26 (37.7%) patients, respectively. Hypertrophied vessels and calcification were detected in 38 (51.2%) and 18 (24.3%) patients, respectively. MRI revealed hypointense to isointense lesions on T1-weighted images, isointense to hyperintense lesions on T2-weighted images, and hyperintense lesions on diffusion-weighted imaging; 9 (75%) and 3 (25%) patients demonstrated homogeneous and heterogeneous enhancement, respectively.

**Conclusion:**

CD often shows well-defined, mildly hypodense or isodense, homogeneous lymph nodules on non-enhanced CT/MRI, with intermediate and marked enhancement on contrast-enhanced CT/MRI. Calcification and hypertrophied vessels may be valuable diagnostic features.

## Background

Castleman disease (CD), also known as giant lymph node hyperplasia, lymphoid hamartoma, or angiofollicular lymph node hyperplasia, is a group of uncommon lymphoproliferative disorders that share common lymph node histological features. The disease was first described in a single case in 1954 [1], followed by a small series of cases in 1956 [2]. The cause of CD remains unclear; however, possible causes include chronic inflammation, lymphoid hamartomatous hyperplasia, and an increase in serum interleukin-6 levels, among others [3]. Most commonly, CD is classified clinically as unicentric CD (UCD) or multicentric CD (MCD) and histologically as hyaline vascular variant (HVV) or plasma cell variant (PCV) [3, 4]. CD occurs throughout the body on surfaces such as the chest [5], neck [6], abdomen, pelvis [7], axilla [8], and on rare occasions the lung [9], parotid gland [10], and pancreas [11].

CD is easily confused with lymphoma or other solid tumors. Knowledge of the imaging features of CD is important to ensure prompt diagnosis and treatment. However, the infrequent occurrence of CD has led to a limited analysis of its imaging characteristics as well as susceptibility to misdiagnosis during diagnostic imaging. Some studies have described the CT and MRI features of CD in various parts of the human body; however, these studies were mainly case reports, review articles, and small retrospective studies [12–16]. Hence the present retrospective study described the CT/MRI findings of CD and correlated them to its histopathologic characteristics.

## Materials and methods

The institutional review board approved this retrospective study; the requirement for informed consent was waived.

### Subjects

We searched the pathology database in our hospital and identified patients diagnosed with CD and treated at our hospital between January 2010 and May 2017. In addition, these patients also had a record of CT or MRI examination that was performed within 1 month before surgery or biopsy. In total, 107 patients with CD were identified, 74 (37 men and 37 women; mean age ± SD, 35 ± 15.2 years,) of which had complete clinical and imaging data and were included in this study. The remaining 33 patients were excluded because of incomplete imaging data. Prior to treatment, 69 patients had CT and 12 had MRI scans; 7 patients had both CT and MRI scans. Plain and contrast-enhanced scans were acquired for all patients.

### Clinical manifestations

The following pre-treatment clinical information, when available, was retrieved from the electronic medical record.

### Pathologic data

All cases were diagnosed histopathologically (surgical resection or biopsy) by a lymphatic pathologist. The lesions were classified as HVV when the lymph node (LN) harbored atrophic follicles with hyalinized vessels and concentric rings of lymphocytes, and if the interfollicular cells were predominantly lymphocytes. The lesions were classified as PCV when the LN architecture was characterized by a paucity of follicular hyaline vessels and accompanied by a marked accumulation of plasma cells in the interfollicular areas [17].

### Imaging

CT was performed using a 64-row MDCT scanner (Brilliance64, Philips Medical Systems) and a dual-source CT system (Somatom Definition Flash, Siemens Healthcare Sector) with the following scan parameters: voltage = 120 kV, amperage = 110–210 mA, rotation time = 0.5 s, detector collimation = 0.625 mm, pitch = 0.8–1.0 and section thickness = 3.0–5.0 mm. A non-ionic contrast medium (75–100 ml; Omnipaque 350, GE Healthcare) was intravenously injected using a power injector at a rate of 3 ml/s. An enhanced scan of multiple sites was performed at our institute starting with the chest, followed by the abdomen, and finally of the neck. With the trigger threshold of the aorta reaching 100 HU, a scan of the thoracic and abdominal regions (arterial portal phase) was performed at the trigger, and the portal vein phase of the abdominal region was performed 35 s after the trigger; the neck region was the last to be scanned.

MRI was performed using a 1.5-T MRI scanner (Siemens Medical Solutions) using the following protocol: neck, axial T1 spin echo (TR/TE, 613/8.4 ms), axial T2 TSE (TR/TE, 6020/87.0 ms), coronal T2 TSE (TR/TE, 3000/39.0 ms), axial T1 gradient echo (TR/TE, 251/2.85 ms), axial/coronal post-gadolinium T1; thorax, axial TRUFI (TR/TE, 295.81/1.16 ms), axial T2 half-Fourier acquisition single-shot TSE (TR/TE, 1000/25.0 ms), axial T2 TSE (TR/TE, 3500/74.0 ms), coronal T2 TSE (TR/TE, 3300/71.0 ms), axial diffusion-weighted imaging (DWI) (TR/TE, 2500/68.0 ms; b value, 0,50, 600 s/mm^2^), axial/coronal post-gadolinium T1; abdomen, axial TRUFI (TR/TE, 4.05/1.8 ms), T2 TSE (TR/TE, 2860/84.0 ms), axial DWI (TR/TE, 2500/68.0 ms; b value, 0,50,600 s/mm^2^), in- and out-of-phase T1, pre-gadolinium and axial/coronal post-gadolinium fat-suppressed T1 VIBE (TR/TE, 5.41/2.39 ms); and pelvis, axial T1 TSE (TR/TE, 550/12.0 ms), axial/coronal T2 TSE (TR/TE, 8830/103.0 ms), sagittal TRUFI (TR/TE, 3.53/1.51 ms), axial DWI (TR/TE, 3500/73.0 ms; b value, 0, 50, 800), and axial/coronal and sagittal post-gadolinium T1 VIBE (TR/TE, 5.41/2.39 ms). All contrast-enhanced MRI examinations were performed with a gadolinium chelate administered at a dose of 0.1 mmol/kg body weight.

### Image analysis

Two abdominal radiologists with 7 and 13 years of experience in imaging diagnosis reviewed the images for consensus at PACS workstations (Syngo-Imaging, version VB36A, Siemens Healthcare, Germany). The following CT features of lesions were analyzed: location, distribution, morphology, margin, longest dimension, homogeneity, calcification, degree, and pattern of enhancement, vascular invasion, hypertrophied vessels, pulmonary lesions, ascites or pleural effusion, and splenomegaly. Morphology was categorized as regular-shaped, irregular or lobulated. The longest dimension was measured in maximum cross section on the transverse axis. The degree of enhancement was categorized as hyper-, iso-, or hypo-attenuating compared to the muscles of the same level on the arterial and portal phases. Enhancement pattern was described as homogeneous and heterogeneous. Splenomegaly was defined as the longest diameter of the spleen > 12 cm in cross-section.

The following features were recorded on the MR images: location, distribution, morphology, margin, longest dimension, homogeneity, degree and pattern of enhancement, vascular invasion, hypertrophied vessels, pulmonary lesions, ascites or pleural effusion, splenomegaly, signal intensity relative to that of muscles on T1- and T2-weighted sequences, signal intensity on DWI and the mean ADC, and presence or absence of enhancement and its pattern.

UCD was defined as an enlargement confined to one anatomical node station, while MCD was defined as an enlargement at more than one node station or the presence of multiple enlarged, separated nodes at one node station.

### Statistical analyses

Data were analyzed using the Statistical Analysis System (SAS) software, Version 9.4 (SAS Institute Inc.). Quantitative variables were described using mean ± SD and categorical data using frequency and percentage in the text and figures. Kolmogorov-Smirnov tests were used to check the normality assumption. Univariate analysis was performed by using the Student’s *t-*test or the Mann-Whitney U test. Categorical data were compared using the Chi-square test or Fisher’s exact test. A value of *P* < 0.05 was considered statistically significant.

## Results

### Clinical manifestations

The clinical characteristics of CD are summarized in Table 1. Most of the patients with UCD had no obvious symptoms upon admission to our hospital; however, masses were found either during a routine physical examination or due to the patient accidentally touching the tumor. All MCD patients showed obvious clinical symptoms. There was no statistical difference in sex and age at the onset between MCD patients and UCD patients. The pathological type of most UCD was HVV, whereas that for most MCD was PCV. Only four of the MCD patients were tested for HIV and their results were negative. No patient was tested for human herpesvirus 8(HHV-8).

**Table 1:** Summary of the Main Clinical Information in Castleman disease (*n* = 74)

	UCD (*n* = 65)	MCD (*n* = 9)	*P*
Age (mean, years)	32.2 ± 14.8	41.8 ± 17.4	0.155
Gender			1
Male	33/51%	4/44.4%	
Female	32/49%	5/55.6%	
Pathological type			0.000
HVV	60/92.3%	1/11.1%	
PCV	5/7.7%	8/88.9%	
Non-symptomatic	56	0	0.000
fever	0	9	0.000
cough	9	9	0.000
thoracic or abdominal pain	0	9	0.000
dyspnea	0	8	0.000
hemoptysis	0	4	0.000
B symptoms^a^	0	3	0.000

*UCD* unicentric Castleman disease, *MCD* multicentric Castleman disease, *HVV* Hyaline vascular variant, *PCV* Plasma cell variant

^a^B symptoms, defined as fever, chills, and/or night sweats; two with emaciation

### Imaging findings

#### Ct

For the 65 patients with UCD, the anatomical distribution of the affected lesions as observed through imaging was as follows: abdominal = 30, thoracic = 22, neck = 10, axillary = 2, and shoulder = 1. Compartmental distribution of the thoracic lesions was as follows: mediastinal = 11, hilar = 10, and cardiophrenic angle = 1. Among the abdominal lesions, 22 and 8 were in the retroperitoneal and peritoneal reflection, respectively. For the 9 patients with MCD, the anatomical distribution of the affected lesions at each involved site is shown in Table 2.

**Table 2:** Breakdown of involved sites in multicentric Castleman disease (*n* = 9)

Sites	Neck	Axillary	Mediastinum	Hilum	Peritoneal	Retroperitoneal	Iliac	Inguinal
Case								
1	+	+	+	-	+	+	+	+
2	+	+	+	-	+	+	+	+
3	+	+	+	+	-	+	-	-
4	+	+	-	-	+	+	+	+
5	-	+	+	-	-	-	-	-
6	+	+	+	+	-	+	+	+
7	+	-	-	-	-	-	-	-
8	-	+	+	+	+	+	+	+
9	+	+	+	+	+	-	+	+
Total	7	8	7	4	5	6	6	6

For the patients with UCD, the mean of the longest lesion diameter was 5.5 ± 2.4 cm (range, 1.5 to 14.0 cm) with the lesions measuring < 5.0 cm in 34 patients and ≥ 5.0 cm in 31 patients. For the patients with MCD, the mean of the longest lesion diameter was 2.3 ± 1.0 cm (range, 1.0 to 4.9 cm). The lesions were significantly larger in patients with UCD than in those with MCD (*p* = 0.000).

**Fig. 1:**
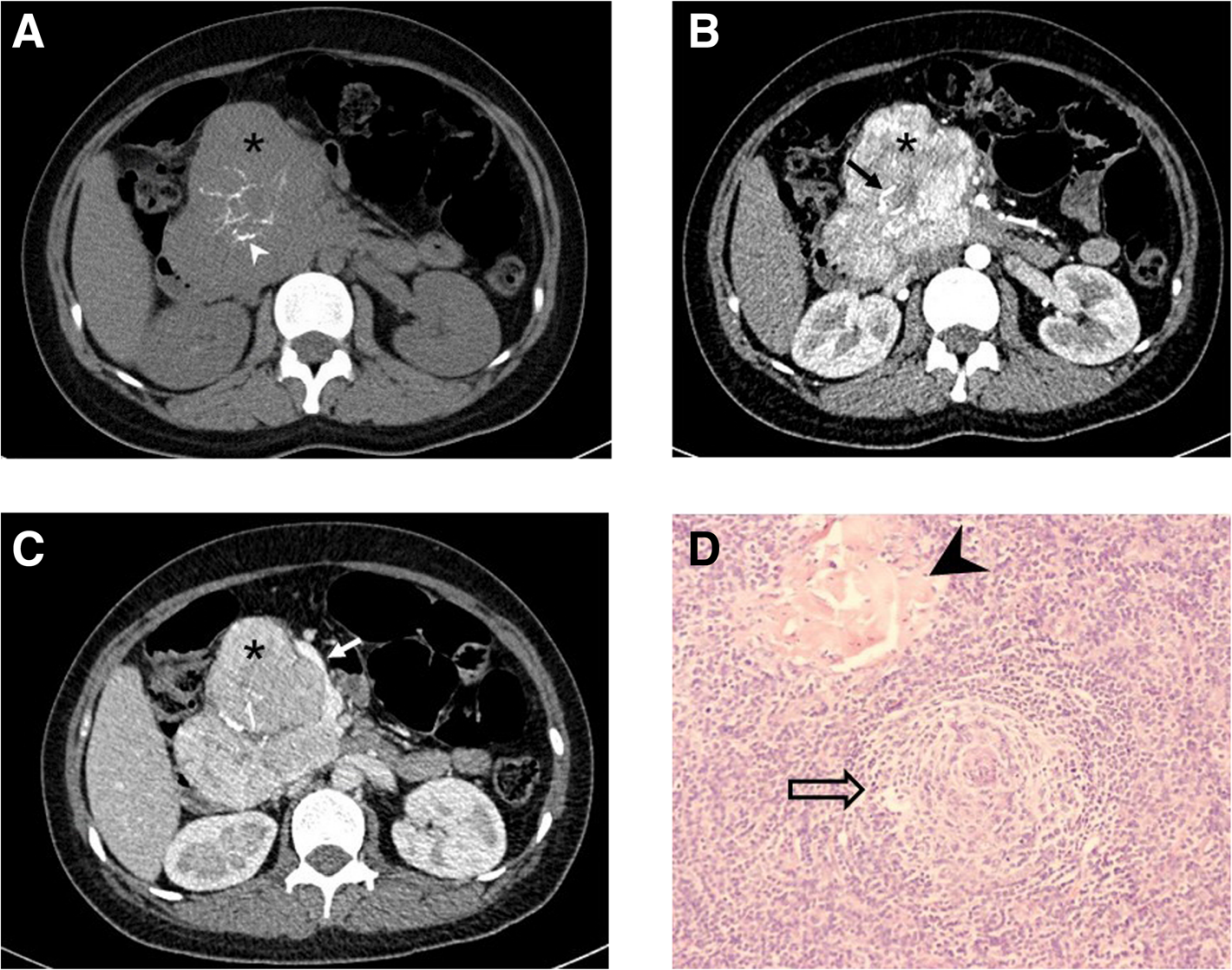
HVV unicentric Castleman disease in a 26-year-old woman. Axial non-enhanced (**a**), arterial phase (**b**), and portal venous phase (**c**) computed tomography images of the abdomen depict a well-defined heterogeneous mass of soft-tissue density (*) with branched calcification at the pancreatic head (arrowhead), which shows progressive enhancement, slightly greater than that of the pancreas. The feeding artery (black arrow) and draining vein (white arrow) can be seen. Photomicrograph (**d**, original magnification, × 200; hematoxylin-eosin [H-E] staining) shows marked vascular proliferation and hyalinization of the abnormal germinal center, with a tight concentric layering of lymphocytes at the periphery of the follicle, resulting in an “onion-skin” appearance (open arrow) and vessel-rich interfollicular stroma (arrowhead). (UCD = unicentric Castleman disease; MCD = multicentric Castleman disease; HVV = Hyaline vascular variant; PCV = Plasma cell variant)

The main CT findings in 69 patients with CD who underwent CT are summarized in Table 3. The morphology of all lesions was regular. On non-enhanced CT images, the lesions appeared hypodense to isodense relative to the skeletal muscle, and all PCV lesions and most HVV lesions were homogeneously dense. The enhancement pattern was homogeneous in 43 (62.3%) patients, including 36 (83.7%) with < 5.0 cm lesions and 7 (16.3%) with ≥5.0 cm lesions. The enhancement pattern was heterogeneous in 26 (37.7%) patients, including 17 (65.4%) with < 5.0 cm lesions and 9 (34.6%) with ≥5.0 cm lesions. Among the 33 patients who underwent abdominal scanning, 18 (54.5%) and 15 (45.5%) demonstrated peak contrast enhancement on portal or arterial phase images, respectively (Fig. 1).

**Table 3:** Summary of the Main Computed Tomography Findings in Castleman disease (*n* = 69)

CT Findings	HVV (*n* = 57)	PCV (*n* = 12)	*P*
Clinical type			0.000
Unicentric	56	4	
Multicentric	1	8	
Margin			0.540
Well Circumscribed	51	12	
Infiltrate into the Surrounding	6	0	
Longest diameter	5.4 ± 2.4 cm (rang: 1.5 ~ 14.0 cm)	2.6 ± 1.7 cm (rang: 1.0 ~ 10.9 cm)	0.000
Homogeneity			0.450
Homogeneous	50	12	
Heterogeneous	7	0	
Pattern of enhancement			0.008
Homogeneous	31	12	
Heterogeneous	26	0	
Degree of enhancement			1
Hyper--	56	12	
Iso-	0	0	
Hypo-	1	0	
Calcifications	18^a^	0	0.057
Punctate	13	0	
Branched	5^a^	0	
Coarse	4^a^	0	
Hypertrophied vessels	38	0	0.000
Feeding artery	26^b^	0	
Draining vein	25^b^	0	
Splenomegaly (*n* = 8)^c^			0.002
Unicentric	1	1	
Multicentric	0	6	

*UCD* unicentric Castleman disease, *MCD* multicentric Castleman disease, *HVV* Hyaline vascular variant; *PCV* Plasma cell variant

^a^In four patients, the CD lesion presented multiple calcified areas with different patterns

^b^13 patients had both the hypertrophied feeding arteries and draining veins

^c^33 patients underwent abdominal scanning, including 24 UCD and 9 MCD

The calcified lesions were distributed in the retroperitoneum (*n* = 9), mesenterium (*n* = 3), mediastinum (*n* = 2), neck (*n* = 2), pulmonary hilum (*n* = 1), and cardiophrenic angle (*n* = 1). Hypertrophied feeding arteries around the lesions were observed in 26 (35.1%) patients (abdominal = 10, thoracic = 9, neck = 7), while enlarged draining veins around the lesions were observed in 25 (33.8%) patients (abdominal = 10, thoracic = 6 and neck = 9). Both hypertrophied feeding arteries and draining veins (abdominal = 4, thoracic = 3, neck = 6) were seen in 13 (17.6%) patients (Fig. 1). All patients with hypertrophied vessels were of the HVV type.

**Fig. 2:**
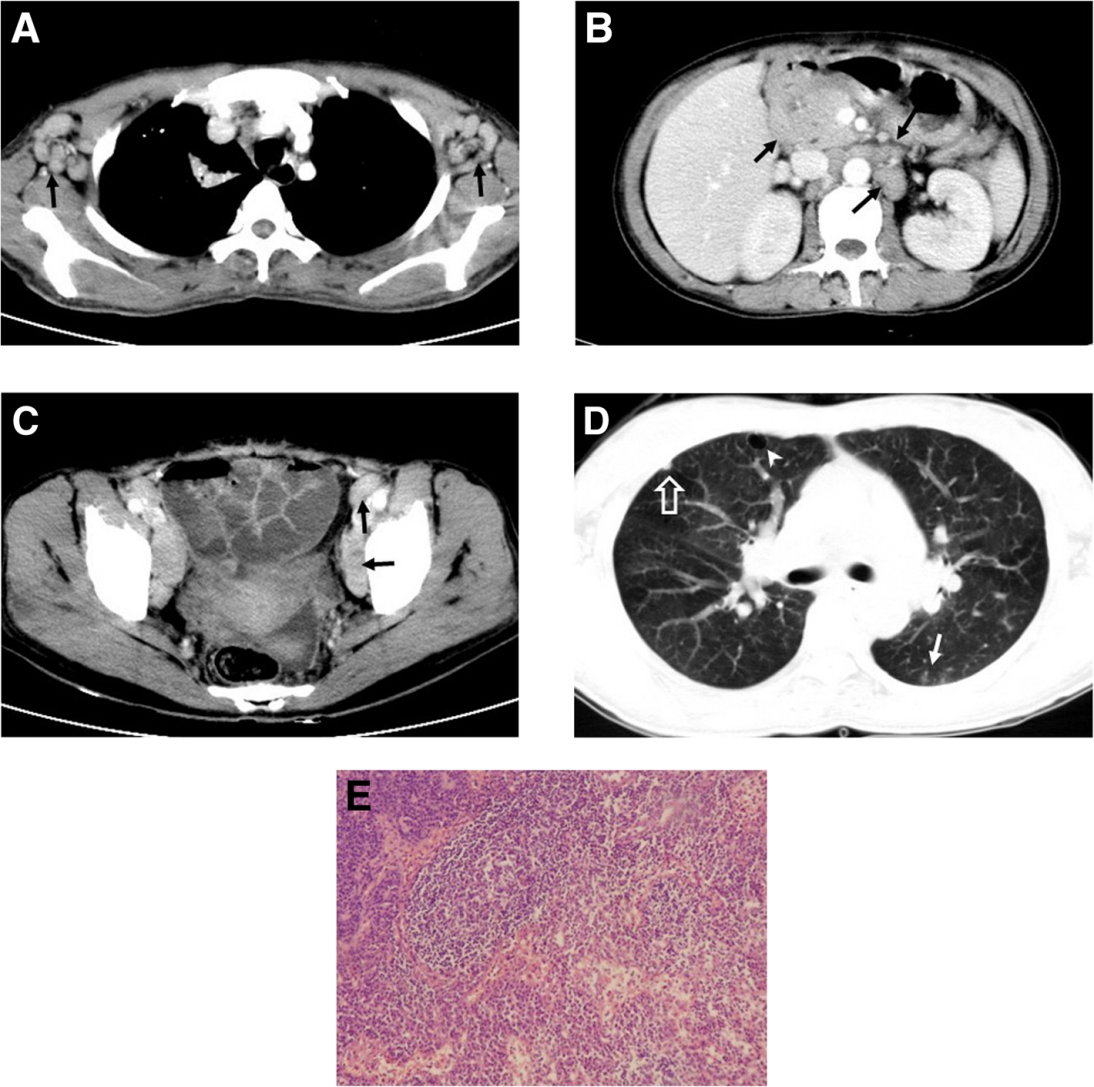
A 43-year-old woman with PCV multicentric Castleman disease. Enhanced computed tomography scan shows multicentric, moderately enhancing, enlarged LNs (arrows) in the bilateral axillae (**a**), abdomen (**b**) and pelvis (**c**). Lung windows (**d**) of the CT scan shows multiple GGOs (arrow), thin-walled cysts (arrowhead), and scattered subpleural nodules (open arrow) with bronchovascular bundle and interlobular septal thickening. Photomicrograph (**e**, original magnification, × 200; hematoxylin-eosin [H-E] stain) shows diffuse plasma cell proliferations in the interfollicular tissue. (LN = lymph node; UCD = unicentric Castleman disease; MCD = multicentric Castleman disease; HVV = Hyaline vascular variant; PCV = Plasma cell variant; GGOs = Ground-glass opacities)

There were two patients with CD involving the lung. In one patient, multiple ground-glass opacities (GGOs), thin-walled cysts, and scattered subpleural nodules with bronchovascular bundle and interlobular septal thickening were observed (Fig. 2). In the other patient, diffuse, small nodules, interlobular septal and bronchovascular bundle thickening, and scattered GGOs were observed.

In addition, two patients with PCV (one with UCD and the other with MCD) had pleural effusion and ascites at presentation. The patient with MCD had moderate bilateral pleural effusion and severe ascites, whereas the patient with UCD had mild to moderate bilateral pleural effusion and ascites. No abnormal pleural thickening, nodules, or enhancement were observed.

### MRI

**Fig. 3:**
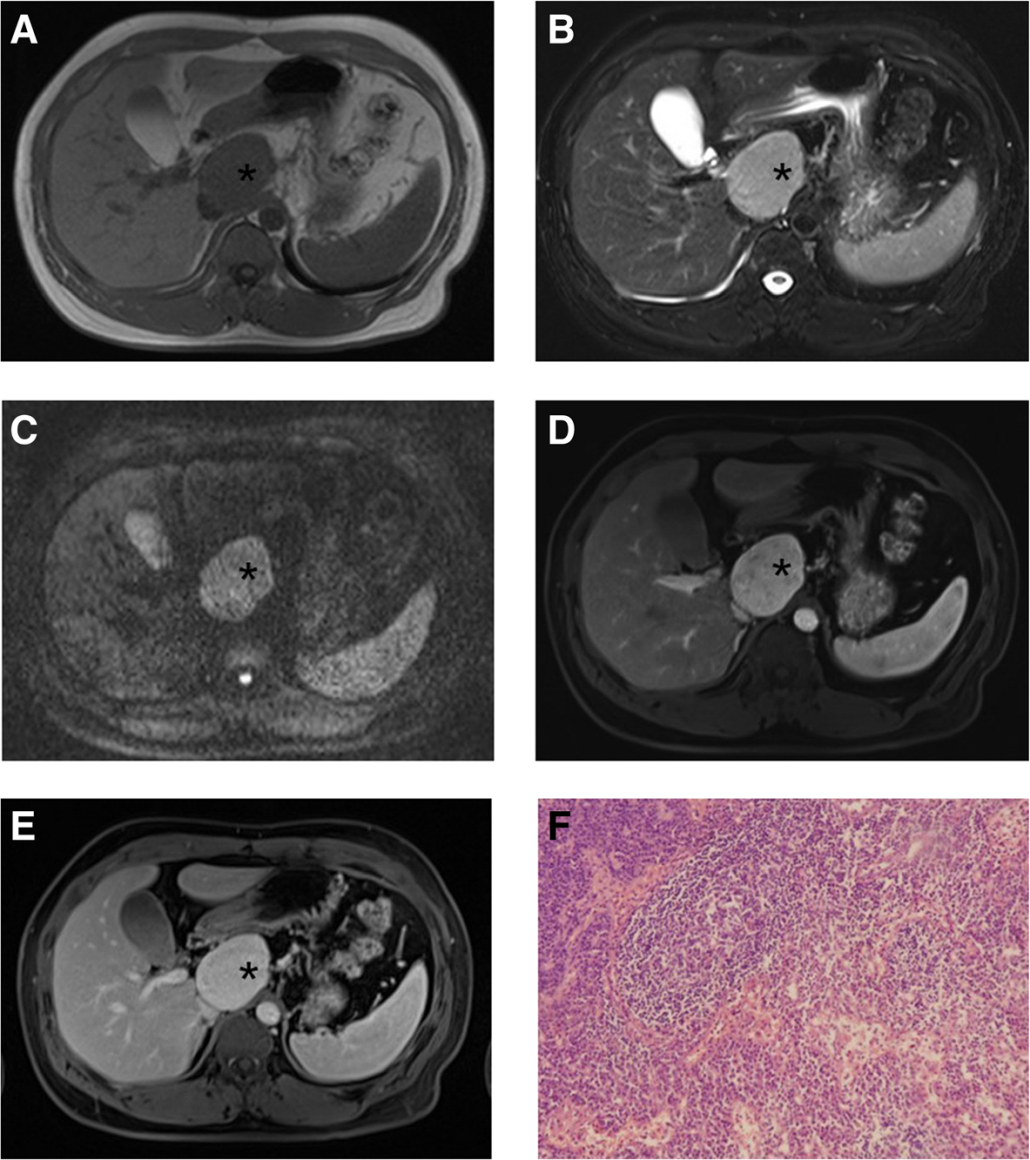
A 36-year-old man with PCV unicentric Castleman disease. T1-weighted image (**a**) and T2-weighted image (**b**) show a mass (*) in the retroperitoneum, well-defined, homogeneous, hypointense on T1, hyperintense on T2 as well as on diffusion-weighted imaging (DWI) b = 600(**c**). After gadolinium contrast injection in the arterial phase (**d**), lesion is hyperintense, followed by sustained hyperintensity in portovenous phase (**e**). Photomicrograph (**f**, original magnification, × 200; hematoxylin-eosin [H-E] stain) shows diffuse plasma cell proliferations in the interfollicular tissue. (UCD = unicentric Castleman disease; MCD = multicentric Castleman disease; HVV = Hyaline vascular variant; PCV = Plasma cell variant)

All 12 patients who underwent MRI had UCD. The main MRI findings of CD are summarized in Table 4. The morphology of all lesions was regular and well-circumscribed. No hypertrophied vessels and splenomegaly were found in these lesions (Fig. 3).

**Table 4:** Summary of Main Magnetic Resonance Imaging Findings in Castleman disease (*n* = 12)

MR Findings	HVV (*n* = 10)	PCV (*n* = 2)	*P*
Longest Diameter	5.5 ± 1.3 cm (rang: 3.7 cm~ 7.8 cm)	6.6 ± 0.3 cm (6.4 cm, 6.8 cm)	0.228
Homogeneity			1
Homogeneous	7	2	
Heterogeneous	3	0	
T1 weighted			1
isointense	5	1	
slight hyperintense	5	1	
T2 weighted			0.167
isointense	0	1	
hyperintense	10	1	
Enhancement Pattern			1
Homogeneous	7	2	
Heterogeneous	3	0	
Enhancement Degree			
Hyper-	10	2	
Diffusion-Weighted Imaging* (*n* = 6)			
hyperintense (b = 600 s/mm^2^)	5 (mean ADC: 1.34 × 10^−3^ mm^2^/s)	1 (mean ADC: 1.09 × 10^−3^ mm^2^/s	

*UCD* unicentric Castleman disease, *MCD* multicentric Castleman disease, *HVV* Hyaline vascular variant; *PCV* Plasma cell variant

*b value = 0, 50, 600 s/mm^2^

#### Follow-up

There was no recurrence during the follow-up period (24–76 months) after surgery in 66 patients. There were 7 patients with PCV MCD that underwent chemotherapy and remained stable (*n* = 3) or in remission (*n* = 4) during the follow-up period of 24–60 months. The patient with MCD exhibited moderate bilateral pleural effusion and severe ascites, which subsequently developed into B-cell lymphoma.

## Discussion

In our study, most patients with UCD had no clinical symptoms, whereas most MCD patients had significant clinical symptoms; most of the UCD histopathological types were HVV, while most of the MCD histopathological subtypes were PCV, similar to previous studies [4, 17, 18]. Most of the lesions encountered in our study were noninvasive masses with well-defined borders. On the non-enhanced CT images, CD classically presented slightly hypodense to isodense lesions, whereas the degree of enhancement varied on contrast-enhanced CT images. The degree of enhancement was not significantly different between the patients with HVV and those with PCV, which is similar to the findings in studies by Hill et al. [14] and Kwon et al. [19] but different from the findings in another study [20]. More than half of the patients observed had hypertrophied vessels on CT images in our study, and all were HVV. Calcification was observed in some patients, moreover, all calcified lesions were observed in patients with HVV UCD, although a previous study also found calcification in PCV CD or MCD [7].

All lesions were hyperintense or isointense relative to the muscle on T1-weighted images and hyperintense or isointense on T2-weighted images. After gadolinium enhancement, all lesions demonstrated homogeneous enhancement. The MRI findings of CD in the present study were consistent with previous findings. DWI was performed for 6 patients and all lesions demonstrated hyperintensity on high b-value imaging; the result was similar to that of the study by Khalil et al. [21] but was higher than that reported by Oida et al. [22].

The lesions were significantly larger in patients with UCD than in those with MCD, probably because most patients with UCD had the HVV subtype, allowing for abundant blood supply. For lesion calcification, we hypothesized that the formation and type of calcification may be closely related to the histopathology of the lesion. A large number of scattered lymphoid follicles were observed in HVV lesions, and there were abundant capillaries and venous hyperplasia and vitreous degeneration in or between the follicles. In this study, the cases with calcified lesions had thickened small vessel walls with narrowing of the lumen in the central region, obvious hyaline degeneration, extensive area hardening and fibrosis, and other degenerative changes; calcium deposition occurred on the basis of these changes. Calcified lesions were distributed along the small blood vessels, and some of those micro-calcifications merged to form larger calcium deposits, which may explain the central distribution of calcification and the morphological features of branches or spots. In a previous study, dramatic enhancement during the arterial phase and decreased enhancement during the portal–venous phase was observed [16]. In addition to this pattern of enhancement on abdominal images, we also found that some lesions had a different pattern of enhancement, that is, continuous enhancement. This pattern of enhancement has not been reported before. Moreover, the hypertrophied vessels of lesions were interesting and important findings. Preoperative evaluation of feeding and draining vessels around lesions is very important since the standard treatment for patients with UCD is complete surgical resection [18, 23]. Preoperative embolotherapy for hypervascular nodal vessels may facilitate uncomplicated surgical resection [24, 25].

In this study, we observed that both UCD and MCD presented some characteristic imaging features. These features may be helpful in clinical practice for the accurate diagnosis of CD instead of biopsy (especially since biopsy is an invasive technique, or may be difficult to conduct in some lesion locations), to assess the effects of follow-up treatment, or to assist image-guided biopsy, etc.

One limitation of our study is that HHV-8 detection tests were not performed; therefore, MCD could not be classified into its subtypes. Further, the number of patients who underwent MRI was small, and the limitation of the equipment resulted in a comparatively low b value. Lastly, although whole-body MRI plays an important role in the assessment of hematological diseases and is a good choice for MCD requiring extensive detection of lesions due to its radiation-free nature, MCD patients in our study could not undergo whole-body MRI examination as it was unavailable at our hospital. Future studies should assess the role of whole-body MRI in the diagnosis of CD.

## Conclusions

In conclusion, the findings of this study suggest that the anatomic site of CD is not specific. CD often shows well-defined, mildly hypodense or isodense, homogeneous nodules or masses on non-enhanced CT/MR images, and intermediate and marked enhancement on contrast-enhanced CT/MR images. The hypertrophied vessels are valuable features. The calcification in affected lesions is not rare and is more commonly observed in HVV UCD.

In future, the differential diagnosis of UCD and other origins of single soft tissue tumors as well as the differential diagnosis of MCD and lymphoma should be investigated.

## Data Availability

The datasets used and/or analyzed during the current study are available from the corresponding author on reasonable request.

## Abbreviations

CD: Castleman disease
HHV-8: Human herpesvirus 8
HVV: Hyaline vascular variant
MCD: Multicentric Castleman disease
PCV: Plasma cell variant
UCD: Unicentric Castleman disease
